# A Comparison of Complications between Open Abdominal Sacrocolpopexy and Laparoscopic Sacrocolpopexy for the Treatment of Vault Prolapse

**DOI:** 10.1155/2013/528636

**Published:** 2013-09-26

**Authors:** Anne-Lotte W. M. Coolen, Anique M. J. van Oudheusden, Hugo W. F. van Eijndhoven, Tim P. F. M. van der Heijden, Rutger A. Stokmans, Ben Willem J. Mol, Marlies Y. Bongers

**Affiliations:** ^1^Department of Gynaecology and Obstetrics, Máxima Medical Centre, De Run 4600, 5500 MB Veldhoven, The Netherlands; ^2^Department of Gynaecology and Obstetrics, Isala Klinieken, Dokter van Heesweg 2, 8025 AB Zwolle, The Netherlands; ^3^Department of Gynaecology and Obstetrics, Zorggroep Twente, Zilvermeeuw 1, 7609 PP Almelo, The Netherlands; ^4^Department of Epidemiology, CAPHRI Research School, Maastricht University, Minderbroedersberg 4-6, 6211 LK Maastrich, The Netherlands; ^5^Department of Gynaecology and Obstetrics, Academic Medical Centre, Meibergdreef 9, 1105 AZ Amsterdam, The Netherlands

## Abstract

*Introduction*. Sacrocolpopexy is a generally applied treatment for vault prolapse which can be performed laparoscopically or by open laparotomy. *Methods*. Between October 2007 and December 2012, we performed a multicenter prospective cohort study in 2 university and 4 teaching hospitals in the Netherlands. We included patients with symptomatic posthysterectomy vaginal vault prolapse requiring surgical treatment, who either had abdominal or laparoscopic sacrocolpopexy. We studied surgery related morbidity, which was divided in pre-, peri-, and postoperative characteristics. *Results*. We studied 85 patients, of whom 42 had open abdominal and 43 laparoscopic sacrocolpopexy. In the laparoscopic sacrocolpopexy group, estimated blood loss was significantly less compared to the abdominal group: 192 mL (±126) versus 77 mL (±182), respectively (*P* ≤ .001). Furthermore, hospital stay was significantly shorter in the laparoscopic group (4.2 days) as compared to the abdominal group (2.4 days) (*P* ≤ .001). The overall complication rate was not significantly different (*P* = .121). However there was a significant difference in favor of the laparoscopic group in peri- and postoperative complications requiring complementary (conservative) treatment and/or extended admittance (RR 0.24 (95%-CI 0.07–0.80), *P* = .009). *Conclusion*. Laparoscopic sacrocolpopexy reduces blood loss and hospital stay as compared to abdominal sacrocolpopexy and generates less procedure related morbidity.

## 1. Introduction

The incidence of posthysterectomy vault prolapse requiring surgery has been estimated at 36 per 10,000 women years [1]. The risk increases cumulatively with years after hysterectomy and increases significantly in women whose initial hysterectomy was performed for genital prolapse [1–3]. In an aging population, the number of women that will seek medical help for a vaginal vault prolapse will increase due to an improved life expectancy and due to the aging population.

Surgery for pelvic organ prolapse, including vaginal vault prolapse, focuses on the restoration of the normal vaginal anatomy and normal bladder and bowel function. To date, a variety of different surgical procedures to correct vaginal vault prolapse have been reported [4]. These reconstructive techniques can principally be divided into vaginal or abdominal procedures. The abdominal approach can be performed open or laparoscopically. According to a Cochrane review on the subject, abdominal sacrocolpopexy is associated with a lower rate of recurrent vault prolapse compared to the vaginal sacrospinous fixation [5]. Success rates of abdominal sacrocolpopexy range between 93% and 99% [6, 7].

The first report of a laparoscopic approach for sacrocolpopexy was written in 1994 [8]. Laparoscopy has potential advantages in terms of reduced morbidity, shorter hospital stay, and convalescence. Surgically, it has been suggested that there is easier access and placement of the mesh down the posterior vaginal wall compared with the open procedure [9]. Previous studies showed less blood loss and a significantly shorter hospital stay in the laparoscopic group [5, 9, 10]. These studies show no significant difference in complication rate; however in the abdominal group more severe complications occurred [9].

All previous studies have shown less morbidity in favor of the laparoscopic method, but prospective comparisons are lacking, specifically to evaluate differences in complication rates between both procedures. We performed a prospective cohort study to compare complication rates of the open abdominal sacrocolpopexy and the laparoscopic sacrocolpopexy.

## 2. Materials and Methods 

We report on patients who underwent surgery for posthysterectomy symptomatic vault prolapse between 2007 and 2012. The study was performed in 4 teaching and 2 academic hospitals in the Netherlands. The study was approved by the ethical committees of all participating hospitals.

The patient population consisted of women with a history of hysterectomy followed by a symptomatic vault prolapse requiring surgical treatment. Patients having a contra-indication for sacrocolpopexy were excluded. Women who had undergone previous abdominal or vaginal vault prolapse surgery were also excluded.

The population of this study exists of prospectively enrolled patients who were eligible for both surgical techniques (laparoscopy and open sacrocolpopexy) at time of inclusion. Part of the patients were placed in a group dependent on their own preference. Other patients participated in randomized clinical trial comparing laparoscopy and open surgery (ISRCT number: NTR3276) which is still enrolling, and were therefore allocated by blind randomization. This randomized trial is not the focus of the current report. All patients gave permission to use their medical data after signing written informed consent. Women received a case number to treat their data anonymously.

### 2.1. Surgical Intervention

Interventions were either abdominal or laparoscopic sacrocolpopexy. Patients received bowel preparation the day before the operation. Prophylactic antibiotics were given preoperatively, for example, metronidazole/cefazolin. As prophylaxis for thromboembolism pre- and postoperatively subcutaneous daltaparine was administered. 

Looking at the design of this surgical intervention, the main goal of sacrocolpopexy is to reconstitute an adequate, safe, safe durable system of support and suspension vagina by replacing the impaired and/or detached native fascial tissue with a synthetic nonabsorbable prosthesis. 

### 2.2. Abdominal Sacrocolpopexy

The abdominal sacrocolpopexy was performed by a laparotomy under general anesthesia. A Pfannenstiel incision was used preferably. One piece of type 1 polypropylene mesh was attached anteriorly and another as far down the posterior vaginal wall as possible. Both meshes were sutured to each other after which the posterior mesh was fixed to the sacrum. The mesh was peritonealised at several points.

### 2.3. Laparoscopic Sacrocolpopexy

The laparoscopic sacrocolpopexy was performed under general anesthesia with four trocars, one for the scope and three side trocars. The procedure was the same as the abdominal procedure. The vaginal vault was elevated with a vaginal probe. The peritoneum was incised laparoscopically by scissors to expose the rectovaginal and vesicovaginal fascia, extending to the sacral promontory. One piece of type 1 polypropylene mesh was attached anteriorly and another as far down the posterior vaginal wall as possible. The mesh was attached to the sacral promontory using staples. The mesh was peritonealised at several points. 

Both procedures were completed with any necessary vaginal operation after the vault suspension had been carried out. All centers used polypropylene meshes, the same sutures (vicryl) to attach the mesh to the vaginal vault and staples for attachment of the mesh to the sacrum. A urethral catheter was left insitu and was removed after 24 hours postoperative or as clinically indicated. Study surgeons were skilled in performing one or both procedures. To exclude a learning curve, they needed to have performed at least twenty-five procedures prior to the start of participation of the study.

Data collection included duration of symptoms, body mass index, pre- or postmenopausal status, use of estrogens, combined prolapse surgery or stress urinary incontinence, procedure time, amount of estimated blood loss and hospital stay, and perioperative complications. Complications were defined as unintended and undesirable events or situations during or because of a medical intervention, which will have (temporary) negative effects on patients well-being. Severe complications were defined as peri- and post-operative complications due to the surgical intervention requiring complementary (conservative) treatment and/or extended admittance. The Dutch complication registration of the NVOG (Dutch Society of Obstetrics and Gynecology) [11] was used to separate minor from major complications. 

Preoperative, at 6 weeks, and 1 year postoperative a pelvic examination was done according to the recommendations of the ICS (POP-Q classification) to evaluate the anatomical results of prolapse repair.

### 2.4. Statistical Analysis

Data were analyzed based on intention to treat principle. To examine differences between groups we used an unpaired *t*-test for continuous variables and a chi-square or, if opportune, Fisher's exact test for dichotomous variables. A *P* value of <0.05 was considered to be statistically significant. The statistical package used was SPSS 20.0. 

## 3. Results

*Baseline Characteristics*. We included 85 patients in our study, of whom 42 had undergone open abdominal sacrocolpopexy and 43 had undergone laparoscopic sacrocolpopexy. Pre- and postoperative morbidity data could be analyzed in all 85 patients.  Table 1 shows the baseline characteristics of the studied population. No significant differences were observed between both groups. Preoperative POP-Q was also not different in both groups (Table 2).

Table 3 shows perioperative data. In the laparoscopic sacrocolpopexy group estimated blood loss was significantly less compared to the abdominal group: 192 mL (±126) versus 77 mL (±182), respectively (*P* ≤ .001). Furthermore, hospital stay was significantly shorter in the laparoscopic group (4.2 days) as compared to the abdominal group (2.4 days) (*P* ≤ .001). Mean operation time in the abdominal group was 118 min, whereas the mean operation time in the laparoscopic procedure was 128 min (*P* = .254). 

In the laparoscopic group 8 (18.6%) patients had one or more complications compared to 14 (33.3%) patients in the abdominal group which was not significantly different (RR 0.558 (95%-CI 0.26–1.19), *P* = .121). We divided complications in peri- and postoperative complications. 

Perioperative complications are listed in Table 4. There were 5 (11.6%) complications in the laparoscopic group, versus 3 (7.1%) in the open abdominal group (*P* = .489). In the laparoscopic group in one case the procedure had to be converted to an open abdominal sacrocolpopexy due to a bleeding coming from the promontory. This bleeding resulted in a total estimated blood loss of 1200 mL. This patient also developed an abdominal wall hematoma around the median laparotomy scar. In another patient a bleeding of the right ovarian artery occurred while fixating the mesh to the promontory. The total estimated blood loss in this case was 200 mL. In the laparoscopic group 2 bladder injuries occurred of which in one case the operation had to be converted to a vaginal procedure. In one patient in the laparoscopic group the mesh had to be fixated to the ventral abdominal wall because of poor visualization and excessive vascularization of the promontory. 

In the open abdominal group one procedure had to be converted to intravaginal slingplasty due to severe adhesions. In one patient in the open abdominal group some of the mesh sutures were put through the bladder, detected and cut by cystoscopy. One other patient had a bladder injury which was repaired directly.

Postoperative complications are also listed in Table 4. Most of the complications were found in the open abdominal group. There were 6 (14.0%) complications in the laparoscopic group, versus 13 (31.0%) in the open abdominal group (*P* = .086). In the laparoscopic group there was one patient with atrial fibrillation and another patient had symptoms of angina pectoris; nevertheless in both cases no cardiac ischemia could be diagnosed. There was one postoperative wound infection of the umbilical trocar incision and an abdominal wall hematoma; however no complementary treatment was needed. 

In the abdominal group one patient died postoperatively because of multiorgan failure due to a sepsis after a bowel perforation. She had a relaparotomy but developed a pneumonia postoperatively and in combination with multiorgan failure and cardiac arrhythmia, this resulted in a fatal complication 5 days postoperative. Two patients had a wound dehiscence which needed to be surgically repaired. One patient developed a pulmonary embolism which required therapeutic anticoagulant therapy. In three patients, which were 7.1% of the patients in the open abdominal group, an ileus was diagnosed. These three patients recovered after conservative treatment. In two patients there was an abdominal hematoma; however no complementary treatment was needed. One patient had temporary a mild delirium during admittance. Another patient had symptoms of dysuria in combination with fever caused by an urinary tract infection and was treated with antibiotics. Postoperatively, we found two patients in each group with urinary retention.

Three (7.0%) patients in the laparoscopic group and 12 (28.6%) patients in the abdominal group had peri- and postoperative complications due to the surgical intervention requiring complementary (conservative) treatment and/or extended admittance which was significantly different (RR 0.24 (95%-CI 0.07–0.80), *P* = .009). When we separated the minor peri- and postoperative complications from the major complications according to the Dutch complication registration of the NVOG (Dutch Society of Obstetrics and Gynecology) [11], there were no major complications in the laparoscopic group and 3 (7.1%) major complications in the abdominal group which is not statistically significant (*P* = .074). Two of these three major complications of the abdominal group are classified as category B because a re-operation was required to repair the wound dehiscence. The third complication was a category D complication since the complication was fatal [11].

Six weeks postoperative there were 16 (18.8%) complications seen which are shown in Table 5. There were 7 (16.7%) complications in the laparoscopic group, versus 9 (21.4%) in the open abdominal group (*P* = .527). In the laparoscopic group seven patients had complications of which five patients (11.6%) had constipation, one patient (2.3%) developed de novo stress urinary incontinence, and another patient (2.3%) had de novo urge incontinence. The complications of the open abdominal sacrocolpopexy group consisted of three patients (7.1%) with de novo stress urinary incontinence, three patients (7.1%) had symptoms of constipation, another two patients (4.8%) had recurrent urinary tract infections and one patient (2.4%) had bothersome pain around the Pfannenstiel incision. Because all these complications could be a result of permanent loss of function, they are classified as category C complications according to the Dutch complication registration of the NVOG [11].

## 4. Discussion

We performed a prospective cohort study with 85 patients who underwent a sacrocolpopexy either abdominally or laparoscopically for vaginal vault prolapse. Morbidity was less in the laparoscopic group. Less blood loss during surgery and a shorter hospital stay were seen in the laparoscopic sacrocolpopexy group. The overall complication rate was not significant different in both groups; however a trend was seen towards less complications in the laparoscopic group. Peri- and postoperative complications, due to the surgical intervention requiring complementary (conservative) treatment and/or extended admittance, was significantly different in favor of the laparoscopic group. No significant difference was seen in operation time.

Both the Cochrane collaboration and the National Institute for Clinical Excellence (NICE, UK) have recommended abdominal sacrocolpopexy with mesh as the optimal surgical treatment for vaginal vault prolapse [12, 13]. Both open and laparoscopic procedures appear equally effective in several case series [10, 14–17]. 

No large studies powered on complications rates of sacrocolpopexy have been done. On the other hand, many studies were done comparing open abdominal hysterectomy to laparoscopic hysterectomy performed for benign diseases. Two large trials of Garry et al. [18] and Maresh et al. [19] showed a significantly higher complication rate in the laparoscopic hysterectomy group. Nevertheless, hospital stay and postoperative pain were less in the laparoscopic group. We found less (severe) complications in the laparoscopic sacrocolpopexy group. Our study showed similar results on morbidity in accordance to previous studies comparing open abdominal to laparoscopic sacrocolpopexy [5, 9, 10]. A randomized trial comparing laparoscopic to abdominal sacrocolpopexy performed by Freeman et al. [9] showed significantly less blood loss (56 versus 240 mL), less mean Hb drop (1.12 versus 2.33 mg/dL), shorter hospital stay (3.2 versus 4.1 days), and less morphine use (16 versus 32 mL) in the laparoscopic group. There was no significant difference in complication rate, but in the abdominal group more severe complications occurred [9]. Our trial also showed more severe complications in the abdominal group. The overall complication rate of our study was not significantly different (*P* = .121). However there was a significant difference in perioperative complications due to the surgical intervention requiring complementary (conservative) treatment and/or extended admittance in favor of the laparoscopic group (*P* = .009). 

Nonrandomized cohort studies of Paraiso et al. [5] and Klauschie et al. [10] also showed less blood loss and a significantly shorter hospital stay in the laparoscopic group [5, 10]. There was no difference in complication rate between both studies [5, 10]. 

The complication rate of the laparoscopic group was 18.6% (8 patients) compared to a complication rate of 33.3% (14 patients) in the abdominal group. In the abdominal group 3 patients (7.1%) of the patients were diagnosed with an ileus postoperative. At six weeks postoperative 5 patients (11.6%) of the laparoscopic group developed defecation problems compared to 3 patients (7.1%) in the abdominal group. In the abdominal group 3 patients (7.1%) developed de novo stress incontinence. Bowel symptoms are also reported in other studies comparing laparoscopic to abdominal sacrocolpopexy [5, 9, 10]. Klauschie et al. [10] described one severe bowel obstruction in the abdominal group. In the study of Paraiso et al. [5] two patients developed an ileus in the abdominal group, small bowel obstructions occurred once in the laparoscopic group and twice in the abdominal group and one patient of the laparoscopic group, had severe constipation postoperatively. Freeman et al. [9] showed de novo urinary incontinence in both groups, mainly in the abdominal group (2 versus 4 patients) [9] which matches to our study results. In contrast with other studies comparing laparoscopic to abdominal sacrocolpopexy [5, 9, 10], no mesh erosion has been seen in our study population. Patients should be informed preoperatively about complications that occur in more than 5%. This means that constipation and urinary incontinence should be added to the counseling prior to a sacrocolpopexy. For the abdominal sacrocolpopexy counseling should also include the risk of ileus. Physicians should screen patients at risk for bowel symptoms and anticipate by prescribing prophylactic laxatives to prevent constipation. 

The complication rate in our study is higher than prescribed in other studies comparing open abdominal to laparoscopic sacrocolpopexy. Differences in complications rates can be explained by our accurate complication registration. All minor and major complications were precisely documented resulting in honest prospective data. In contrast with other studies all complications are mentioned and prescribed, even when the complication was temporary and did not affect the operation, time of admission, or treatment. The study of Paraiso et al. [5] and Freeman et al. [9] prescribes only severe or permanent complications. 

This study has a few limitations. Some patients were randomized as part of a larger trial that is not the focus of the current report. Not all included patients were randomized. We added a prospective cohort group of women with a history of hysterectomy followed by a symptomatic vault prolapse requiring surgical treatment. Only patients who were eligible for both surgical options were asked for our prospective cohort group. However we did not expect study bias by including this prospective group to our randomized group because we focused on procedure-related morbidity. Patient's or physician's preference does not affect procedure morbidity. Furthermore, no significant differences in baseline characteristics were found.

Several studies showed that laparoscopic sacrocolpopexy is attended with less morbidity and less severe complications [5, 9, 10]. Our results also showed severe complications in the open abdominal group, including a fatal bowel perforation. 

## 5. Conclusions 

Laparoscopic sacrocolpopexy seems to be related to less procedure-related morbidity compared to open abdominal sacrocolpopexy concerning less estimated blood loss, hospital, and severe complications. Laparoscopic sacrocolpopexy is a safer treatment for vaginal vault prolapse compared to abdominal sacrocolpopexy whereas this technique does not prolong the operation. 

## Figures and Tables

**Table 1:** Baseline characteristics (ITT-analysis).

	Laparoscopic sacrocolpopexy *N* = 45	Open abdominal sacrocolpopexy *N* = 44	*P* value
Age (years)			
Median (IQR)	66.2 (61.2–72.7)	67.6 (64.1–73.6)	*.746 *
Body mass index (kg/m^2^)			
Median (IQR)	25.4 (22.5–27.4)	25.6 (23.9–28.3)	*.805 *
Parity (*n/m*)			*.311 *
0	2.4% (1/42)	0.0% (0/40)	
1	7.1% (3/42)	7.5% (3/40)	
2	42.9% (18/42)	45.0% (18/40)	
3	40.5% (17/42)	25.0% (10/40)	
≥4	7.1% (3/42)	22.5% (9/40)	
Menopausal status (*n/m*)			*.543 *
Premenopausal	2.3% (1/44)	4.7% (2/43)	
Postmenopausal	97.7% (43/44)	95.3% (41/43)	
Incontinence (*n/m*)			*.453 *
None	59.5% (25/42)	51.2% (21/41)	
Stress	4.8% (2/42)	7.3% (3/41)	
Urge	14.3% (6/42)	7.3% (3/41)	
Combined	21.4% (9/42)	34.1% (14/41)	
Estrogens use (*n/m*)			*.122 *
None	94.3% (33/35)	82.4% (28/34)	
Local/Systemic	5.7% (2/35)	17.6% (6/34)	
History of gynecological surgery (*n/m*)			*.143 *
TVH only	38.1% (16/42)	27.5% (11/40)	
TVH and ACR	9.5% (4/42)	5.0% (2/40)	
TVH and PCR	2.4% (1/42)	20.0% (8/40)	
TVH and ACR/PCR	28.6% (12/42)	12.5% (5/40)	
TVH, later ACR and mesh	2.4% (1/42)	0.0% (0/40)	
TAH, ACR and later PCR	0.0% (0/42)	2.5% (1/40)	
TAH only	9.5% (4/42)	27.5% (11/40)	
TAH and PCR	2.4% (1/42)	2.5% (1/40)	
Laparoscopic hysterectomy	4.8% (2/42)	0.0% (0/40)	
Supracervical hysterectomy	2.4% (1/42)	0.0% (0/40)	

TVH: transvaginal hysterectomy, TAH: total abdominal hysterectomy.

ACR: anterior colporrhaphy, PCR: posterior colporrhaphy.

**Table 2:** Preoperative POP-Q.

	Open abdominal sacrocolpopexy *N* = 42	Laparoscopic sacrocolpopexy *N* = 43	*P* value
Aa	−0.3 ± 1.8 (−3–3)	−0.5 ± 1.6 (−3–3)	*.667 *
Ba	0.6 ± 2.8 (−5–8)	0.6 ± 2.3 (−3–4)	*.766 *
C	−0.3 ± 4.2 (−8–10)	1.0 ± 2.3 (−6–6)	*.092 *

GH	3.7 ± 0.8 (3–5)	3.7 ± 0.8 (2–5)	*.361 *
PB	3.1 ± 0.5 (2–4)	2.5 ± 0.9 (0–3)	*.102 *
TVL	7.9 ± 1.5 (4–10)	7.7 ± 1.3 (6–11)	*.374 *

Ap	−0.7 ± 1.7 (−3–3)	−1.2 ± 1.9 (−3–3)	*.142 *
Bp	0.1 ± 2.7 (−4–8)	−0.4 ± 2.5 (−3–4)	*.444 *
D	—	—	—

**Table 3:** Perioperative data (PP-analysis).

	Laparoscopic sacrocolpopexy *N* = 43	Open abdominal sacrocolpopexy *N* = 42	*P*value
Operative time (minutes)			
Median (IQR)	120 (110–140)	120 (105–132)	*.884 *
Estimated blood loss (mL)			
Median (IQR)	50 (10–100)	200 (100–250)	*<.001 *
Hospital stay (days)			
Median (IQR)	2 (2-3)	4 (3–5)	*<.001 *

**Table 4:** Patients with perioperative and postoperative complications (PP-analysis).

	Laparoscopic sacrocolpopexy *N* = 43	Open abdominal sacrocolpopexy *N* = 42	*P* value
Perioperative complications			*.479 *
One or more complications	11.1% (5)	7.1% (3)	
Severe adhesions	—	2.4% (1)	
Bladder lesion	4.7% (2)	4.8% (2)	
Bleeding	4.7% (2)	—	
Alternative mesh fixation	2.3% (1)	—	
Postoperative complications			*.086 *
One or more complications	14.0% (6)	28.6% (12)	
Fatal bowel perforation	—	2.4% (1)	
Wound dehiscence	—	4.8% (2)	
Pulmonary embolism	—	2.4% (1)	
Ileus	—	7.1% (3)	
Abdominal wall hematoma	2.3% (1)	4.8% (2)	
Cardiac complication	4.7% (2)	—	
Delirium	—	2.4% (1)	
Wound infection	2.3% (1)	—	
Urinary tract infection	—	2.4% (1)	
Urinary retention	4.7% (2)	4.8% (2)	
One or more treated complications*	7.0% (3)	28.6% (12)	*.009 *
One or more major complications**	—	7.1% (3)	*.074 *

*Complications due to the surgical intervention requiring complementary (conservative) treatment and/or extended admittance.

**According to the Dutch complication registration of the NVOG [11].

**Table 5:** Patients with complications 6 weeks postoperative (PP-analysis).

	Laparoscopic sacrocolpopexy *N* = 43	Open abdominal sacrocolpopexy *N* = 42	*P* value
Complications 6 weeks postoperative			*.527 *
One or more complications	16.7% (7)	21.4% (9)	
Constipation	11.6% (5)	7.1% (3)	
De novo stress incontinence	2.3% (1)	7.1% (3)	
De novo urinary incontinence	2.3% (1)	—	
Recurrent urinary tract infections	—	4.8% (2)	
Bothersome pain around incision	—	2.4% (1)	
